# Genome-Wide Identification and Cold Stress Response Mechanism of Barley Di19 Gene Family

**DOI:** 10.3390/biology14050508

**Published:** 2025-05-06

**Authors:** Wenbo Chai, Chao Yuan, Shufen Li, Hanyuan Xu, Qing Zhu, Hongtao Li, Wei Ji, Jun Wang

**Affiliations:** Lianyungang Academy of Agricultural Sciences, Lianyungang 222006, China; 13215605136@163.com (W.C.); 18361482151@163.com (C.Y.); 15061354746@163.com (S.L.); xuhanyuan1988@126.com (H.X.); zhuqingqz@163.com (Q.Z.); hongtaoli1987@163.com (H.L.); jiwei100500@163.com (W.J.)

**Keywords:** barley, cold stress, Di19 gene family, gene expression

## Abstract

The Di19 (Drought-induced 19) gene family encodes Cys2/His2-type zinc finger proteins that are known to be involved in plant responses to various abiotic stresses, including drought, salinity, and temperature extremes. However, little is known about their roles in barley (*Hordeum vulgare*), particularly in cold stress adaptation. This study aimed to conduct a comprehensive genome-wide analysis of the barley genome to identify Di19 gene family members and examine their expression patterns under cold stress, providing theoretical support for stress-resistant barley breeding. By aligning Di19 gene sequences from *Arabidopsis* and rice and using BLASTp, seven *HvDi19* genes were identified in barley. Bioinformatics analysis revealed that all members contain a conserved Cys2/His2-type zinc finger domain and nuclear localization signals. Phylogenetic analysis grouped the *HvDi19* genes into four subfamilies, with three homologous gene pairs, and Ka/Ks analysis indicated strong purifying selection. Tissue-specific expression analysis showed significant variation in *HvDi19* expression across barley organs. Under cold stress, different barley varieties exhibited distinct *HvDi19* gene expression profiles: for instance, *HvDi19-1* was downregulated in cold-tolerant varieties, whereas *HvDi19-7* showed increased expression in a cold-tolerant mutant, suggesting their potential roles in modulating cold response. These findings reveal the evolutionary conservation and cold-responsive expression characteristics of the *HvDi19* gene family, laying a foundation for future functional studies. The results also provide important molecular resources for the genetic improvement of cold tolerance in barley, contributing to the development of stress-resilient crop varieties under climate change.

## Simple Summary

The Di19 (Drought-induced 19) gene family plays a crucial role in plant responses to environmental stresses. In this study, we conducted a genome-wide identification of the Di19 gene family in barley (*Hordeum vulgare*) and analyzed its expression patterns under cold stress. A total of seven *HvDi19* genes were identified, all of which contained a conserved Cys2/His2-type zinc finger domain and nuclear localization signals. Phylogenetic analysis classified these genes into four subfamilies, and evolutionary analysis revealed strong purifying selection. Tissue-specific expression analysis showed distinct expression patterns among different barley tissues and varieties under cold stress. Our findings provide insights into the evolutionary conservation and functional characteristics of the *HvDi19* gene family, contributing to a better understanding of its role in cold stress response and its potential application in breeding barley for stress tolerance.

## 1. Introduction

Barley (*Hordeum vulgare* L.) is one of the most important cereal crops globally. Due to its strong tolerance to salinity, drought, and cold, it is widely cultivated in arid, semi-arid, and high-altitude regions, where it plays a crucial role in ensuring food security and sustainable agricultural development [1,2]. However, low-temperature stress severely impacts barley’s growth and development, leading to physiological metabolic disorders and reduced photosynthetic capacity, ultimately affecting its yield and quality [3,4]. Although barley is widely grown in high-latitude and high-altitude regions because of its cold tolerance, low temperature remains a major environmental constraint. Therefore, investigating the potential role of the HvDi19 gene family in the barley cold stress response is of significant importance for understanding barley’s cold stress adaptation mechanisms and promoting molecular breeding. Studying barley’s cold-tolerance-related genes and their regulatory mechanisms holds both theoretical and practical value for improving barley’s environmental adaptability and cold-resistant breeding. Plants rely on a complex signaling regulatory network to respond to stress, with transcription factors serving as key regulatory elements, playing vital roles in signal transduction and gene expression regulation [5]. The Di19 (Drought-induced 19) protein is a type of transcription factor closely associated with abiotic stress responses. Previous studies have confirmed that these proteins are involved in responses to drought, salt, and other environmental stresses in model plants such as rice and *Arabidopsis*. However, their functions in barley remain largely unknown. A detailed investigation of the composition, evolutionary characteristics, and expression patterns of the barley *Di19* gene family under cold stress will provide valuable insights into their regulatory roles. Understanding the role of the HvDi19 gene family in low-temperature adaptation will help reveal the molecular basis of barley’s cold tolerance and provide theoretical support and candidate gene resources for cold-resistant breeding.

Transcription factors are proteins that specifically bind to DNA and regulate downstream gene expression, playing a crucial role in plant growth, development, and stress responses [6,7]. They typically contain several conserved domains, including a DNA-binding domain, a transcriptional regulatory domain, a signal molecule interaction domain, and a nuclear localization signal [8,9]. Under abiotic stress conditions, plants can detect environmental changes through a signaling system and activate specific transcription factors through cascade signaling pathways. These transcription factors recognize and bind cis-regulatory elements (such as ABRE, DRE/CRT, etc.), regulating the expression of downstream genes to mediate a series of physiological and biochemical responses, including osmotic regulation, antioxidant protection, and the maintenance of cellular structure stability, thereby enhancing the plant’s adaptability to stress [10,11,12]. The Di19 gene family is an evolutionarily conserved Cys₂/His₂-type zinc finger protein, which plays an important role in plant stress responses [13]. These family proteins are typically localized in the nucleus and possess highly conserved DPLISF and FVQDLVL motifs at the C-terminus, which are thought to be associated with their DNA-binding activity and transcriptional regulatory functions [14]. Previous studies have shown that Di19 gene family members exhibit significant transcriptional regulatory functions under various abiotic stress conditions. For example, in rice (*Oryza sativa*), *OsDi19-4* expression is significantly upregulated under drought stress, and its overexpression enhances drought tolerance in rice while affecting the expression of genes related to the antioxidant system [15]. In *Arabidopsis*, the *Di19-1* overexpression line exhibits stronger drought resistance and affects the regulation of the ABA signaling pathway [16]. Additionally, in wheat (*Triticum aestivum*), *TaDi19A* shows an upregulation in response to osmotic stress, salt stress, and cold stress, suggesting its potential role in multi-stress responses [17]. Although the *Di19* gene family has been functionally characterized in several plant species, the systematic genome-wide identification and functional analysis of this family in barley remain limited. Furthermore, the role of *HvDi19* genes in the cold stress response remains unclear. There is a pressing need for comprehensive studies to investigate their expression patterns under cold stress and elucidate their regulatory networks, which will help to reveal their functional contributions to cold tolerance in barley.

Based on the conserved domains of the *Arabidopsis AtDi19* genes, this study performed a genome-wide identification of *HvDi19* family members in barley for the first time. By integrating bioinformatics, transcriptomic analyses, and cold stress treatments, we systematically characterized the gene structure, evolutionary relationships, and expression profiles of the *HvDi19* family. In contrast to previous research, that primarily focused on model species or other crops, this study targets barley, a cold-tolerant cereal, to explore the potential functions of *HvDi19* genes in cold stress adaptation, thereby filling a significant knowledge gap in this field. The findings provide not only a theoretical foundation for functional studies of the *HvDi19* gene family, but also valuable genetic resources for the molecular breeding of cold-tolerant barley cultivars.

The objectives of this study are threefold: (1) to conduct a genome-wide identification and structural analysis of *HvDi19* genes, including their gene structure and conserved motif characterization; (2) to investigate their phylogenetic relationships and expression profiles in different tissues and under cold stress conditions; (3) to analyze genotype-specific expression differences among barley varieties to explore potential regulatory mechanisms. The results of this study will advance our understanding of the *Di19* gene family’s roles in cold stress responses and provide essential molecular markers and theoretical support for breeding programs aimed at enhancing cold tolerance in barley.

## 2. Materials and Methods

### 2.1. Experimental Materials

The barley varieties used in this study included Yangnongpi 5 (Yang 5), provided by Yangzhou University, and Liansimai 1 (Si 1), Gang Pi No. 3 (Gang 3), its mutant strain (Gang 3 Mut), and Gang Pi No. 6 (Gang 6), all independently bred by the Lianyungang Academy of Agricultural Sciences.

The main reagents used were SYBR High-Sensitivity qPCR SuperMix (catalog number: E099) and NovoScript^®^ Plus All-in-One 1st Strand cDNA Synthesis SuperMix (gDNA Purge) (catalog number: E047), both purchased from NovoStart.

The main equipment included a fluorescent quantitative PCR instrument (QuantStudio 6 Flex real-time PCR system (Applied Biosystems, Thermo Fisher Scientific, Waltham, MA, USA) and a benchtop refrigerated centrifuge (X3R centrifuge, Thermo Fisher Scientific, USA).

### 2.2. Gene Family Member Identification

Based on the protein sequences of Di19 gene family members from *Arabidopsis thaliana* and *Oryza sativa*, homologous alignments were performed in the Phytozome database version 13.1 (https://phytozome-next.jgi.doe.gov/, accessed on 20 November 2024) to identify Di19 protein sequences from *Hordeum vulgare* L. and *Brachypodium distachyon*. Subsequently, the candidate genes were subjected to conserved domain validation using the Pfam database version 35.0 (http://pfam.xfam.org/, accessed on 22 November 2024) and the SMART database version 11 (https://smart.embl.de/, accessed on 22 November 2024) databases to confirm the presence of the typical Di19 zinc-binding domain.

### 2.3. Protein Basic Feature Analysis

Chromosome localization information for *HvDi19* gene family members was obtained from the Phytozome database, and based on their relative positions on the chromosomes, the *HvDi19* genes were renamed. The basic physicochemical properties of the HvDi19 proteins, including amino acid sequence length, molecular weight, theoretical isoelectric point (pI), instability index, aliphatic index, and grand average of hydropathicity (GRAVY) value, were analyzed using the ProtParam tool from Expasy (https://web.expasy.org/protparam/, accessed on 25 November 2024) to evaluate protein stability, thermal stability, and hydrophilic/hydrophobic characteristics. the SignalP software version 6.0 (https://services.healthtech.dtu.dk/service.php?SignalP, accessed on 25 November 2024) was used to predict whether the HvDi19 proteins contained signal peptides in order to assess their potential subcellular localization and secretion characteristics. the TMHMM software version 2.0 (https://services.healthtech.dtu.dk/service.php?TMHMM, accessed on 25 November 2024) was employed to predict transmembrane domains in the proteins and identify *HvDi19* family members with potential membrane-binding characteristics. The hydrophilicity and hydrophobicity distribution patterns of the proteins were analyzed using ProtScale (https://web.expasy.org/protscale/, accessed on 25 November 2024) to reveal their structural stability and functional features. the MEME Suite version 5.5.0 (https://meme-suite.org/meme/tools/meme, accessed on 25 November 2024) was used to perform a conserved motif analysis of the HvDi19 protein family to identify potential functionally important domains, which were further annotated with functional domains from the Pfam database version 35.0 (https://www.ebi.ac.uk/interpro/pfam/, accessed on 25 November 2024), providing a basis for protein functional analysis. The three-dimensional (3D) structures of the HvDi19 proteins were predicted using the SWISS-MODEL tool (https://swissmodel.expasy.org/, accessed on 25 November 2024), facilitating the further exploration of their potential spatial conformations and functional mechanisms.

### 2.4. Phylogenetic Tree Construction and Gene Structure Analysis

The protein sequences were aligned using MEGA 7.0 software, and a phylogenetic tree was constructed using the Neighbor-Joining (NJ) method. A total of 1000 bootstrap replicates were performed to assess the reliability of the phylogenetic relationships. Gene pairs with genetic similarity greater than 90% were identified, and their non-synonymous substitution rate (Ka) and synonymous substitution rate (Ks) were calculated using the Ka/Ks_Calculator to evaluate evolutionary selection pressure. A sliding window analysis was performed usingthe DnaSP software, version 6 (https://www.ub.edu/dnasp, accessed on 28 November 2024). Protein motif distributions were predicted using the MEME Suite version 5.5.0 (https://meme-suite.org/meme/, accessed on 28 November 2024), with the maximum number of motifs set to 10 and the motif length ranging from 6 to 60 amino acids. Gene structure diagrams were drawn using the GSDS software version 2.0 (http://gsds.gao-lab.org/, accessed on 28 November 2024) by integrating coding sequences (CDSs) with genomic sequences, thereby visually presenting the exon–intron distribution features.

### 2.5. Tissue Expression Pattern Analysis

To explore the spatiotemporal expression pattern of the *HvDi19* genes, transcriptome sequencing data for barley were downloaded from the Expression Atlas database (https://www.ebi.ac.uk/gxa/experiments, accessed on 12 December 2024) (datasets E-MTAB-2809 and E-MTAB-4634). The FPKM (Fragments Per Kilobase of transcript per Million mapped reads) values or Fold Change values for *HvDi19* genes in different tissues and under stress conditions were extracted. Expression heatmaps were generated using the TBtools software version 1.098 (https://github.com/CJ-Chen/TBtools-II, accessed on 3 January 2025) to reveal the expression trends of the genes.

### 2.6. Cold Stress Treatment

For cold stress analysis, seedlings of different barley varieties at the three-leaf stage were exposed to −4 °C for 48 h in a growth chamber. Leaf samples were collected immediately after treatment in order to perform RNA extraction and gene expression analysis.

### 2.7. Total RNA Extraction and Quantitative Real-Time PCR (qRT-PCR) Detection

For this study, barley varieties independently bred by the Lianyungang Academy of Agricultural Sciences were selected, with Yangnong Pi 5 (Yang5) used as the control group. The experimental group included Lian Feed Barley No. 1 (Feed1), Gangpi No. 3 (Gang3), a mutant strain of Gangpi No. 3 (Gang3 mutant), and Gangpi No. 6 (Gang6). After cold stress treatment, barley leaf samples were collected for fluorescence quantitative analysis, with three biological replicates. RNA was extracted according to the instructions for the Yisheng MolPure^®^ Plant RNA Kit. The extracted RNA was stored at −80 °C for long-term preservation. cDNA synthesis was performed using the NovoScript^®^ Plus All-in-One 1st Strand cDNA Synthesis SuperMix (gDNA Purge) kit, following the manufacturer’s instructions. The total reaction volume was 20 μL, with the reaction conditions set as 50 °C for 15 min and 85 °C for 5 s, followed by cooling on ice and short-term storage at −20 °C. The primer sequences used for the qRT-PCR are listed in Table 1.

## 3. Results

### 3.1. Identification and Analysis of Di19 Gene Family Members

Based on the Di19 protein sequence of *Arabidopsis thaliana*, a homologous search was performed in the Phytozome database version 13.1 (https://phytozome-next.jgi.doe.gov/, accessed on 20 November 2024) to identify Di19 protein family members in *Hordeum vulgare* L. (barley) and *Brachypodium distachyon* (short-awned grass). Subsequently, the protein sequences obtained from the search were validated for their domain structures using the Pfam database version 35.0 **(**http://pfam.xfam.org/, accessed on 22 November 2024) and the SMART database version 11 (https://smart.embl.de/, accessed on 22 November 2024) databases to confirm the presence of the typical Di19 zinc-binding domain. The results indicated that the barley genome contains a total of seven Di19 genes, which were named *HvDi19-1* to *HvDi19-7* based on their chromosomal locations. Similarly, seven Di19 genes were identified in the *B. distachyon* genome, named *BdDi19-1* to *BdDi19-7*. Chromosomal localization analysis showed that the Di19 genes in barley are primarily located on chromosomes 1, 3, and 5. The protein lengths encoded by these genes range from 105 to 252 amino acids, with molecular weights ranging from 24,977.96 Da to 26,356.00 Da, and isoelectric points (pIs) spanning from 4.44 to 8.87. In contrast, the Di19 protein sequences of *B. distachyon* range mainly from 206 to 236 amino acids, with molecular weights between 23,343.72 Da and 28,396.68 Da, and isoelectric points ranging from 4.47 to 6.45 (Table 2).

### 3.2. Phylogenetic Analysis of Di19 Family Proteins in Barley and Brachypodium Distachyon

**Figure 1:**
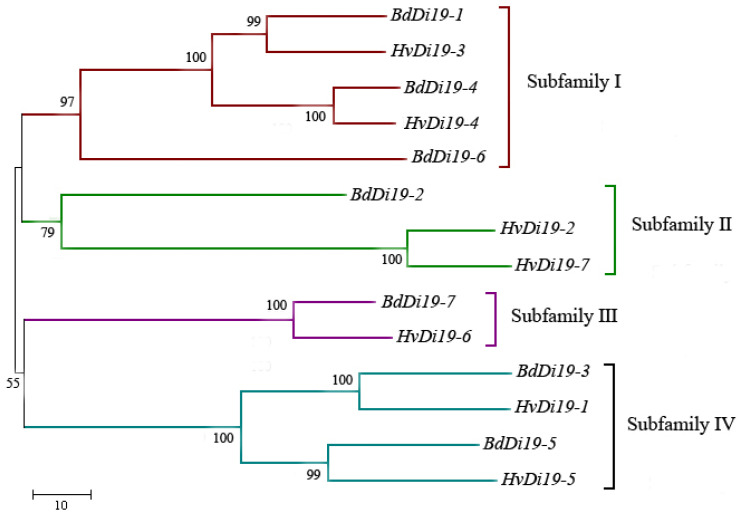
Phylogenetic tree of gene family. Bd: *Brachypodium distachyon*; Hv: *Hordeum vulgare*. The tree was divided into four subfamilies designated I to IV.

To analyze the evolutionary relationships of the Di19 protein family in *Hordeum vulgare* L. (barley), a cereal crop, and *Brachypodium distachyon*, a model grass species closely related to barley, the Di19 protein sequences were aligned using MEGA 7.0 software and a phylogenetic tree was constructed (Figure 1). This selection allowed for a detailed comparison within the closely related monocot species and provided insight into the lineage-specific evolution of the Di19 gene family in grasses. The analysis revealed that the Di19 family can be divided into four subfamily branches based on evolutionary relationships. Subfamily I includes five members, namely *BdDi19-1*, *HvDi19-3*, *BdDi19-4*, *HvDi19-4*, and *BdDi19-6*; subfamily II contains only two barley genes, *HvDi19-2* and *HvDi19-7*; subfamily III consists of one barley gene and one *Brachypodium distachyon* gene; and subfamily IV comprises four genes, including two barley genes and two *Brachypodium distachyon* genes.

**Figure 2:**
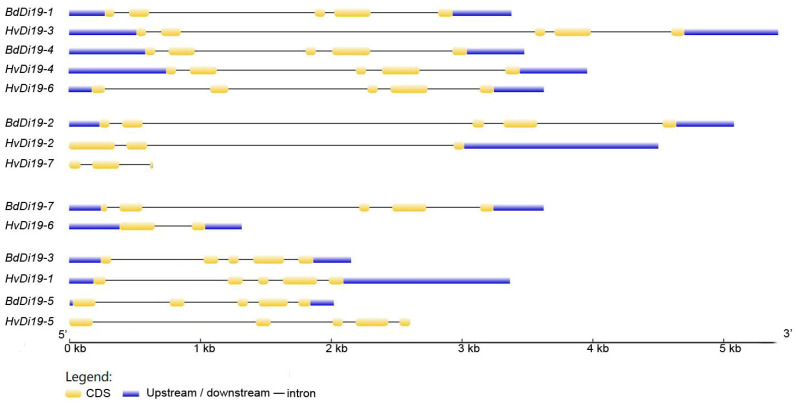
intron/exon gene structure.

To further validate the accuracy of the phylogenetic tree and explore the structural diversity of the Di19 genes, we analyzed their intron–exon structure (Figure 2). The results indicate that all Di19 family genes contain introns, but the number of introns varies significantly between subfamilies. Specifically, members of subfamily I and subfamily IV contain four introns each; *HvDi19-2* and *HvDi19-7* in subfamily II each contain two introns; and *HvDi19-6* in subfamily III contains only one intron. Notably, all the Di19 genes in *Brachypodium distachyon* contain four introns, while the number of introns in the barley Di19 genes ranges from one to four. This suggests that the barley Di19 genes underwent intron loss during evolution, resulting in species-specific gene structures.

### 3.3. Conserved Domains and Three-Dimensional (3D) Structure Analysis of HvDi19 Gene Family

**Figure 3:**
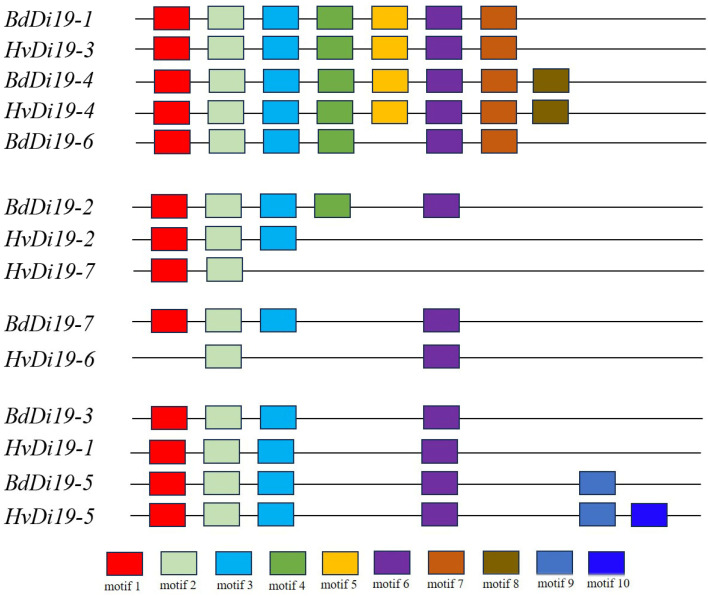
Motifs of Di19 protein structure.

**Figure 4:**
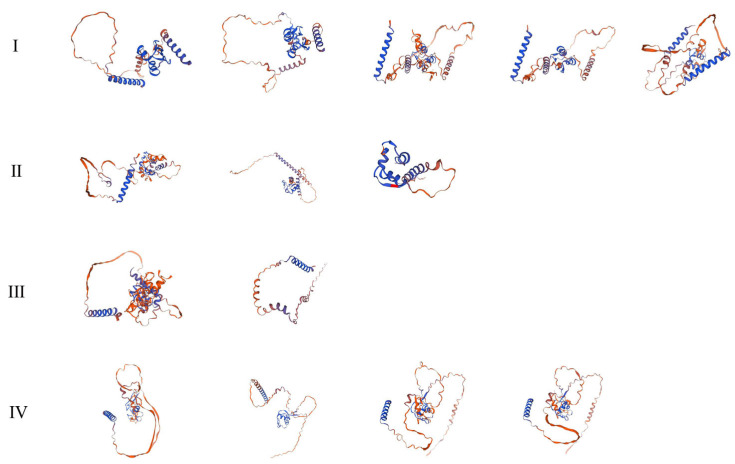
The 3D structure of the proteins. I to IV means the gene family was divided into four subfamilies.

To explore the conserved structural features of the *HvDi19* gene family, MEME software was used to analyze the conserved motifs of 14 Di19 protein sequences (Figure 3). This analysis identified 10 conserved motifs (motif 1 to motif 10) within the Di19 gene family. All the Di19 genes contained these conserved motifs, indicating the high conservation of this family. Motif 1, which is located at the N-terminus of the protein, contains 41 amino acids and constitutes the Di19 zinc-binding domain, the core functional domain of the family. Members of the family generally exhibit similar motif compositions, with motif 1 and motif 2 present in nearly all members. Motif 5, motif 7, and motif 8 are specific to subfamily I, while motif 9 and motif 10 are found exclusively in subfamily IV. The gene structure diagrams and motif distributions further support the phylogenetic classification, suggesting that the HvDi19 gene family developed subfamily-specific motif combinations during evolution. Additionally, to analyze the spatial conformation of the HvDi19s proteins, we used the Phyre2 server for 3D structure prediction and categorized the Di19 proteins of barley and *Brachypodium distachyon* into four groups based on their phylogenetic relationships (Figure 4). The results showed that the 3D structure of Di19 family proteins is relatively simple, which is consistent with their short amino acid sequence characteristics. Their protein structure is mainly composed of random coils, which play a key role in protein function regulation and conformational changes. Typically, conserved amino acid sequences form stable, conserved protein structures, which are responsible for specific biological functions. HvDi19 family proteins not only retain specific conserved domains but also exhibit a certain degree of structural differentiation among different subfamilies. This characteristic may contribute to functional diversity and enable these proteins to perform diverse roles in plant physiological processes.

### 3.4. Evolutionary Selection Analysis of Genes

**Figure 5:**
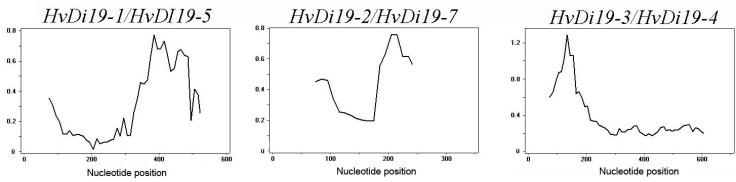
Sliding window analysis.

To further explore the selective pressure on Di19 gene family replication, we calculated the synonymous substitution rate (Ks) and nonsynonymous substitution rate (Ka) of gene pairs and, further, analyzed the Ka/Ks ratio (Table 3). Here, Ka represents nonsynonymous substitutions, Ks represents synonymous substitutions, and Ka/Ks ratio is widely used to measure the genetic evolution rate and reflect the selective pressure on the genes. According to natural selection theory, Ka/Ks > 1 indicates positive selection, Ka/Ks = 1 indicates neutral selection, and Ka/Ks < 1 suggests purifying selection. The results show that, in the barley Di19 gene family, there are three pairs of homologous genes and their Ka/Ks ratios are all less than 1, indicating that these genes primarily undergo purifying selection, with mutations suppressed and sequence evolution occurring at a slower rate. To further estimate the time of gene duplication events, we used the formula T = Ks/(2λ) × 10⁶ Mya (λ = 6.56 × 10⁻⁹) to calculate the Ks values of the three gene pairs, which ranged from 0.2512 to 1.5807. The corresponding duplication event occurred approximately 5.95 to 37.41 million years ago (Mya), suggesting that these genes have undergone stable selective pressure over a long evolutionary period. Further Ka/Ks sliding window analysis (Figure 5) revealed that almost all homologous gene pairs had Ka/Ks ratios of less than 1 across all gene loci, further confirming that the Di19 gene family mainly undergoes purifying selection, and its function has likely remained relatively stable during evolution.

### 3.5. Gene Tissue Expression Pattern Analysis

**Figure 6:**
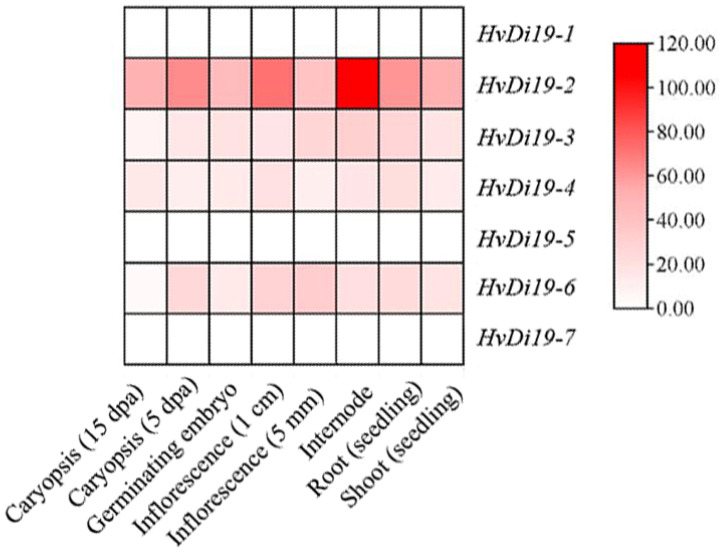
Relative expression profiles of HvDi19 family in different tissues.

Based on transcriptome sequencing data (not under cold stress), as shown in Figure 6, this study analyzed the expression patterns of the *HvDi19* gene family in eight different tissues or developmental stages of barley (Morex), including germinating embryo, seedling shoot, seedling root, internode, inflorescence (5 mm and 1.0 cm), and caryopsis (5 days post-anthesis, dpa, and 15 dpa). The results revealed significant differences in the expression levels of the seven *HvDi19* family members across various tissues. Among them, *HvDi19-1*, *HvDi19-5*, and *HvDi19-7* exhibited very low expression levels in all tissues, suggesting that these genes may have weak functionality or are regulated under specific conditions during barley growth and development. In contrast, *HvDi19-2* displayed relatively high expression levels in multiple tissues, particularly in the internode, where its expression was highest, suggesting that it may play an important role in regulating barley plant height. These results provide a foundation for further elucidating the functions of the *HvDi19* genes and their roles in barley growth and development.

### 3.6. Expression of Di19 Genes in Different Varieties

**Figure 7:**
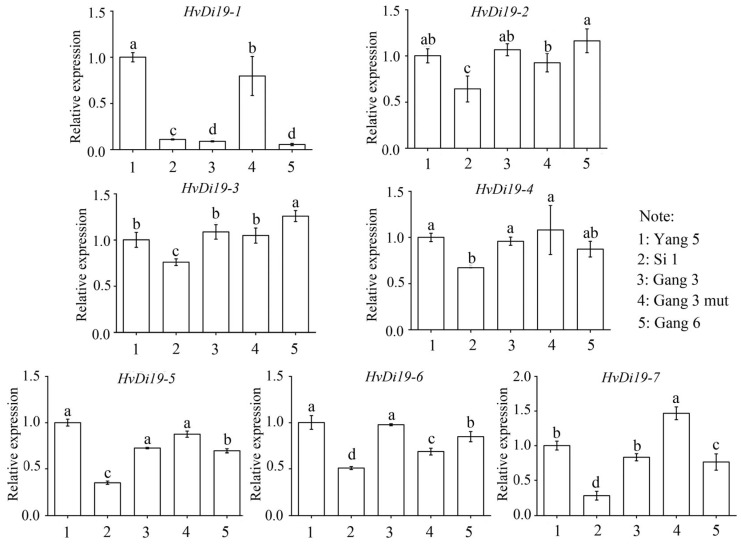
Expression of Di19 genes in different barley varieties under cold stress. Different lowercase letters indicate significant differences in different treatments *p* < 0.05.

**Figure 8:**
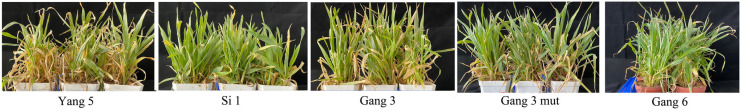
The phenotypes of barley under cold stress. Liansimai 1 (Si 1), Gang Pi No. 3 (Gang 3), its mutant strain (Gang 3 Mut), and Gang Pi No. 6 (Gang 6).

To further analyze the expression levels of the Di19 gene family in different barley varieties, we subjected seedlings to an artificial low-temperature treatment (−4 °C for 48 h) and then measured the gene expression levels in leaf samples using qRT-PCR. The experimental results (Figure 7) showed that the cold resistance of the linkage barley was significantly higher than that of Yangnong Pilsner No. 5 (*p* < 0.05). For these barley varieties, Yangnong Pilsner No. 5 (Yang 5) was used as the control group and Lian Si Mai No. 1 (Si 1), Gang Pilsner No. 3 (Gang 3), a Gang Pilsner No. 3 mutant (Gang 3 mut), and Gang Pilsner No. 6 (Gang 6) were used as the experimental groups for sampling and analysis. The results indicated significant differences in the expression levels of the Di19 genes among the different barley varieties, suggesting that the expression of Di19 genes is significantly affected by cold stress. After cold treatment, the expression of the *Di19-1* gene significantly decreased in Lian Si Mai No. 1, Gang Pilsner No. 3, and Gang Pilsner No. 6 (*p* < 0.05), indicating that the *Di19-1* gene may play a role in barley’s cold stress response. Corresponding to their cold resistance phenotypes (Figure 8), the expression of Di19 genes in Lian Si Mai No. 1 was consistently lower than in the control variety, Yangnong Pilsner No. 5, with these genes being downregulated under cold stress, potentially participating in the negative regulation of barley’s cold resistance. On the other hand, the *HvDi19-7* gene showed higher expression levels in the Gang Pilsner No. 3 mutant, suggesting that this gene may be involved in the positive regulation of barley’s cold resistance. The expression profiles under controlled cold treatment conditions provide valuable insights into the varietal differences in cold stress response.

## 4. Discussion

### 4.1. Conserved Features and Evolutionary Characteristics of Di19 Gene Family

The Di19 gene family is widely distributed among various plants and plays a key regulatory role in plant responses to abiotic stresses [18,19]. Previous studies have shown that Di19 family members are closely related to plant growth hormone signaling pathways and responses to stress factors such as drought and salinity [13,14]. For example, in *Arabidopsis*, the *Di19-3* gene acts as a component of auxin signaling, participating in the regulation of drought and salt stress responses through its interaction with the *IAA14* gene [20,21,22]. In maize, the overexpression of the *ZmDi19-1* gene significantly enhances salt stress tolerance [23]. Similarly, in rice, the *Di19-4* gene regulates the ABA (abscisic acid) signaling pathway by positively regulating the downstream gene *OsCDPK14*, thereby enhancing the plant’s stress resistance [24]. These findings suggest that the Di19 gene family responds to abiotic stress in various plants through different mechanisms, demonstrating its significant role in plant stress resistance. However, the specific functions and mechanisms of Di19 family members vary significantly across different plant species, indicating that the function of Di19 genes in each species may be regulated by genomic backgrounds and species-specific factors [13,14]. For instance, in *Arabidopsis*, Di19 family genes play different roles in drought and salt stress responses, with *AtDi19* acting as a transcription factor to modulate pathogenesis-related gene expression under drought stress [25]. In contrast, in rice, *OsDi19-4* enhances drought resistance by improving reactive oxygen species (ROS) scavenging capacity [15], while in maize, *ZmDi19* genes are implicated in salt stress tolerance through ABA-dependent signaling pathways. These findings highlight that the mechanisms of Di19 gene action may be regulated through distinct signaling pathways tailored to the physiological needs of each species. Therefore, understanding the diversity and functional differences in Di19 genes across different plants is crucial for revealing their universal and specific roles in plant stress adaptation [26].

In this study, we performed a comprehensive identification of the Di19 gene family members in *Hordeum vulgare* and *Brachypodium distachyon* through systematic genomics analysis. Our results showed that the Di19 gene family members in barley and *Brachypodium distachyon* exhibit a high degree of structural conservation, particularly in the presence of zinc finger domains and nuclear localization signals, indicating that Di19 family genes may play similar roles in the stress responses of these plants [25]. Furthermore, by conducting homologous sequence searches and using databases such as Pfam and SMART, we successfully identified multiple Di19 family members and found that these members could be classified into four subfamilies, consistent with previous classifications in other monocots [14]. This classification provides a foundation for further research into the specific roles of different subfamily members in plant growth, development, and stress responses. Phylogenetic analysis revealed a close evolutionary relationship between Di19 family members in barley and *Brachypodium distachyon*, suggesting that these two species may have similar functional requirements in the evolutionary process of Di19 genes. Additionally, we found certain differences in the structure and sequence of genes within the same subfamily, which may be related to the specific functions of these genes in plant stress responses [27,28]. This finding suggests that the function of Di19 family genes is not only related to their conserved structures but may also be influenced by species-specific evolutionary pressures [26]. Our study provides a systematic analysis of the distribution and evolution of the Di19 gene family in *Hordeum vulgare* and *Brachypodium distachyon*, shedding light on the potential functions of this family in plant stress responses.

### 4.2. Structural Diversity and Selective Pressure of Di19 Genes

At the structural and evolutionary levels, this study revealed that the Di19 gene family exhibits both diversity and conservation in conserved motifs and intron structures. The analysis of conserved motifs showed that, although there are some sequence variations in the Di19 gene family among different plant species, they retain a highly conserved zinc finger domain. The Cys2/His2-type zinc finger structure is one of the characteristic features of the Di19 family, involved in DNA binding and transcriptional regulation [29,30]. By comparing the Di19 genes of different species, we found that this domain remains highly conserved in barley, suggesting that it may play an important functional role in the plant’s stress responses. Further analysis of selective pressure revealed that the evolution of the barley Di19 gene family is primarily driven by purifying selection. The Ka/Ks ratio for these genes is less than 1, indicating that they have undergone strong purifying selection during evolution [31]. The presence of purifying selection not only ensures the stability of Di19 genes, but may also be closely related to their core roles in plant growth and development. This finding suggests that the functional role of the Di19 gene family in plant stress responses has been maintained and optimized through natural selection, further validating its importance in plant adaptation to adverse environments, such as drought and low temperatures [32]. Additionally, sliding window analysis revealed the structural relationships between homologous genes. The degree of structural and sequence variation among different genes provides flexibility for the Di19 family during the evolutionary process. For example, there are certain differences in the lengths of introns and the arrangement of exons among Di19 family members, which may be closely related to their specific functions in different plant species and their adaptability to stress responses [14]. The existence of these structural differences could be a key reason why the Di19 gene family adapts to various environmental stress conditions and participates in different physiological processes [33]. In protein structure analysis, Di19 family proteins mainly adopt a disordered coil conformation, which aligns with their relatively short amino acid sequences. Disordered coil structures generally exhibit high flexibility and adaptability, enabling them to perform different functions under various environmental conditions [34,35]. This structural feature may enable Di19 proteins to have greater adaptability in plant stress responses, particularly under environmental conditions such as low temperatures, drought, or salt stress, allowing them to adjust their functions according to changes in stress signals. Future studies analyzing the 3D structure of these proteins and their interactions with stress response pathways will help us to further understand the functional diversity of the Di19 gene family.

### 4.3. Expression Patterns of Di19 Genes and Stress Responses

In barley, the expression patterns of *HvDi19* gene family members vary significantly across different tissues and developmental stages, indicating that these genes may play important roles in plant growth and development [36]. Expression profile analysis revealed that *Di19-1*, *Di19-5*, and *Di19-7* exhibited low expression levels in multiple tissues, suggesting that these genes may play key roles in specific physiological processes and have notable tissue specificity. For instance, the low expression of *Di19-1* and *Di19-7* may be associated with their negative regulatory roles in plant stress responses [13]. In contrast, the *Di19-2* gene was highly expressed in several tissues, especially in the internodes, where its expression level was the highest. This may be closely related to the regulation of barley plant height. The high expression of *Di19-2* may influence the plant’s growth rate and height, thus participating in the regulation of plant morphological development. Future research should further explore the specific functions and mechanisms of this gene in morphological regulation. In terms of stress response, previous studies have shown that, under cold stress, the expression of the *TaDi19A* gene in wheat peaks after 2 h, indicating a strong rapid response to cold conditions [37]. Additionally, in cotton, *GhDi19-1* and *GhDi19-2* proteins interact with downstream protein CDPKs to regulate salt stress and ABA signaling pathways [16]. In soybean, the *GmDi19-5* gene plays a negative regulatory role in stress responses [38]. This study further analyzed the expression patterns of HvDi19 genes in different barley varieties under cold stress. The results indicated significant variation in the expression of Di19 gene family members among barley varieties. For example, under cold stress, *Di19-7* was significantly upregulated in the Gangpi 3 variety, while it was downregulated in the Liansi Mai 1 variety. This variation suggests that the regulatory roles of Di19 genes may be genotype-dependent. Under cold stress conditions, Di19 genes may regulate plant adaptation to low temperatures through interactions with other stress response genes. This differential expression pattern further reveals the potential role of Di19 genes in cold stress response, particularly contributing to variety-specific cold tolerance. The regulatory effects of cold stress on Di19 genes provide a new perspective for understanding the adaptation mechanisms of different barley varieties under low-temperature stress. Future research should combine transcriptomic, metabolomic, and epigenetic analyses to explore the specific mechanisms of Di19 genes in cold stress responses.

The observed expression patterns of *HvDi19* genes under cold stress suggest their potential roles in cold response regulation. For example, the downregulation of *HvDi19-1* in cold-tolerant varieties may indicate a negative regulatory role, while the upregulation of *HvDi19-7* in the Gang 3 mutant points to a potential positive role in enhancing cold tolerance. While further physiological validation is required, these gene expression differences under uniform cold stress conditions provide preliminary molecular evidence for varietal divergence in cold stress adaptation.

Genotypic differences may affect the expression patterns of Di19 genes, thereby influencing plant stress tolerance. To further reveal the role of Di19 genes in barley’s adaptation to stress, future research can deploy gene editing technologies, such as CRISPR/Cas9, to knock out or overexpress specific Di19 genes and investigate their functions under different stress conditions. This study not only provides new insights into the functions of Di19 genes in different varieties and under stress conditions, but also offers theoretical support for genetic improvement and stress resistance breeding in barley. By thoroughly analyzing the functions and regulatory networks of the Di19 gene family, it is expected that more stress-tolerant crop varieties will be developed in the future, enhancing the stability and sustainability of agricultural production.

In future studies, we plan to perform a functional validation of the key *HvDi19* genes identified in this study using VIGS and other genetic approaches in order to elucidate their specific roles in barley’s cold stress tolerance mechanisms.

## 5. Conclusions

This study systematically identified and analyzed the members of the Di19 gene family in barley and their expression characteristics in response to cold stress for the first time. The results showed that the *HvDi19* gene family in barley has undergone strong purifying selection during evolution, retaining highly conserved Cys2/His2-type zinc finger domains and typical nuclear localization signals, which suggest their important regulatory functions in the cell nucleus. Further analysis revealed significant expression differences in the *HvDi19* gene family under cold stress conditions, especially *HvDi19-1* and *HvDi19-7*, which may function as negative and positive regulators, respectively, modulating barley’s cold stress tolerance. This finding provides new molecular clues for the mechanism of cold stress tolerance in barley, and offers potential molecular targets for future cold tolerance breeding. Future research should further explore the specific regulatory mechanisms of the *HvDi19* gene family in cold stress responses to promote its practical application in crop genetic improvement.

## Figures and Tables

**Table 1:** Primer sequence of RT-PCR.

Genes	Forward Primer	Downstream Primer
*HORVU1Hr1G059310*	GCGAGGATTTCGATTTCGTAGG	GCGAGGATTTCGATTTCGTAGG
*HORVU1Hr1G087100*	GCCTTAGCTTCCCAACATTAGA	CCTTGTGAGGGACAAGTCAATA
*HORVU3Hr1G061690*	GGTTGCGCAGATTCGTTATTC	ATGACTATGACCGCCTCCTA
*HORVU1Hr1G003540*	CCTCAGATGATCAAAGGCTGAA	CATGAGCATCTGCTTCACAAAC
*HORVU5Hr1G051150*	TGTGGCGAGGATTTCGACTT	TTAGAAGATAGGGCGAGGCG
*HORVU3Hr1G114280*	CAGGAGGAACACTGCTTTGA	GCGAGTGTTGGAATCTGAAATG
*HORVU5Hr1G023560*	ATCCTCTACTCTCCTCCTTTGT	GCTCCTCAGCACTTTCTTCT
*Actin*	GAGAGGTTACTCCTTCACAACC	GTCTCCAGCTCCTGTTCATAAT

**Table 2:** Gene information.

Gene Name	Gene ID	Amino Acid Number (aa)	Isoelectric Point (pI)	Molecular Weight (MW, Da)	Subcellular Localization	Chromosomal Location
*HvDi19-1*	*HORVU1Hr1G003540*	218	4.79	24,654.31	Nucleus	1H: 7841380..7844750
*HvDi19-2*	*HORVU1Hr1G059310*	208	8.87	22,854.45	Nucleus	1H: 432516342..432520847
*HvDi19-3*	*HORVU1Hr1G087100*	225	5.55	24,977.96	Nucleus	1H: 538689465..538694886
*HvDi19-4*	*HORVU3Hr1G061690*	252	4.74	28,414.8	Nucleus	3H: 469768134..469772099
*HvDi19-5*	*HORVU3Hr1G114280*	233	5.05	26,356.6	Nucleus	3H: 690566983..690569591
*HvDi19-6*	*HORVU5Hr1G023560*	123	4.84	13,933.46	Nucleus	5H: 123880698..123882018
*HvDi19-7*	*HORVU5Hr1G051150*	105	4.44	11,323.24	Nucleus	5H: 399422454..399423095
*BdDi19-1*	*Bradi2g16670*	230	5.41	25,672.55	Nucleus	Bd2: 14640232..14643614
*BdDi19-2*	*Bradi2g28040*	228	5.11	25,078.11	Nucleus	Bd2: 27262827..27267910
*BdDi19-3*	*Bradi2g39370*	206	4.64	23,343.72	Nucleus	Bd2: 39192968..39195124
*BdDi19-4*	*Bradi2g46240*	251	4.61	28,396.68	Nucleus	Bd2: 46434335..46437815
*BdDi19-5*	*Bradi2g61990*	224	5.69	25,592.64	Nucleus	Bd2: 58637326..58639346
*BdDi19-6*	*Bradi3g11400*	236	6.45	26,711.83	Nucleus	Bd3: 9857561..9861195
*BdDi19-7*	*Bradi4g05020*	220	4.47	24,890.41	Nucleus	Bd4: 4097406..4101034

**Table 3:** Analysis of Ka/Ks.

Paralogous Pairs	Ks	Ka	Ka/Ks	Duplication Date (Mya)	Duplication Type
*HvDi19-1–HvDi19-5*	1.5807	0.4730	0.2973	37.41	segmental
*HvDi19-2–HvDi19-7*	0.2512	0.1590	0.6330	5.95	segmental
*HvDi19-3–HvDi19-4*	0.5796	0.1744	0.3009	13.72	segmental

## Data Availability

The original contributions presented in the study are included in the article; further inquiries can be directed to the corresponding authors.
